# Pesticide Residues and Metabolites in Greek Honey and Pollen: Bees and Human Health Risk Assessment

**DOI:** 10.3390/foods12040706

**Published:** 2023-02-06

**Authors:** Konstantinos M. Kasiotis, Effrosyni Zafeiraki, Electra Manea-Karga, Pelagia Anastasiadou, Kyriaki Machera

**Affiliations:** Laboratory of Pesticides’ Toxicology, Department of Pesticides Control and Phytopharmacy, Benaki Phytopathological Institute, 145 61 Kifissia, Greece

**Keywords:** pesticides, metabolites, LC-MS/MS, GC-MS/MS, honey, pollen, risk assessment

## Abstract

Background: Bees encounter a plethora of environmental contaminants during nectar and pollen collection from plants. Consequently, after their entrance into the beehives, the transfer of numerous pollutants to apicultural products is inevitable. Methods: In this context, during the period of 2015–2020, 109 samples of honey, pollen, and beebread were sampled and analyzed for the determination of pesticides and their metabolites. More than 130 analytes were investigated in each sample by applying two validated multiresidue methods (HPLC-ESI-MS/MS and GC-MS/MS). Results: Until the end of 2020, 40 determinations were reported in honey, resulting in a 26% positive to at least one active substance. The concentrations of pesticides ranged from 1.3 ng/g to 785 ng/g honey. For seven active substances in honey and pollen, maximum residue limits (MRLs) exceedances were observed. Coumaphos, imidacloprid, acetamiprid, amitraz metabolites (DMF and DMPF), and tau-fluvalinate were the predominant compounds detected in honey, while several pyrethroids such as λ-cyhalothrin, cypermethrin, and cyfluthrin were also found. Pollen and beebread, as expected, accumulated a higher number of active substances and metabolites (32 in total), exhibiting almost double the number of detections. Conclusions: Although the above findings verify the occurrence of numerous pesticide and metabolite residues in both honey and pollen, the human risk assessment in the majority of the cases does not raise any concerns, and the same applies to bee risk assessment.

## 1. Introduction

Honey and apicultural products such as pollen, beebread, propolis, and royal jelly have been consumed since antiquity [1] and used in pharmaceutical products and supplements worldwide with broad approval of their beneficial effect on human health [2,3,4,5]. Therefore, they should be free from contaminants linked to undesired health effects that also reduce their quality and commercial value. Recent reports designate that the EU is only 60% honey self-sufficient, with an apparent negative equilibrium of imports/exports [6]. Therefore, it relies on imports to a large extent. Hence, the need to increase honey and related apiculture commodities production in the EU is apparent. Such an increase should be accompanied by the quality management of apiculture activity. A major category of contaminants is pesticides, considering that plant protection products (PPPs) are indispensable and broadly applied for the protection of cultivated crops. Evidently, pesticides are strongly related to bees and apicultural commodities since plants and flowers, as the major pollen and nectar resources, can contain such substances. Exposure of bees to contaminants not only affects honey and other apicultural products’ quality [7] but also can have negative effects on their health, even at concentrations found in environmental compartments [8,9]. In order to ensure the quality and safety of apicultural products, such as food, with the main focus on honey, well-organized monitoring programs and sustainable management are necessary, combined with powerful and fully validated analytical methods making use of the most recent technologies in the area. Hence, analytical methods have been developed, validated, and applied by a plethora of research groups for the detection of pesticides and metabolites [10,11,12], other organic pollutants, pyrrolizidine alkaloids [13], and heavy metals [14,15]. The presented work, as a continuation of previous work of our research group [11], aims to provide an overview of the pesticides and metabolites occurrence in honey, pollen, and beebread samples during the period of 2015–2020 in Greece and add further data on the contribution of pesticide residues to the overall chemical burden related to food consumption, human and bee health.

## 2. Materials and Methods

### 2.1. Samples Collection

**Figure 1:**
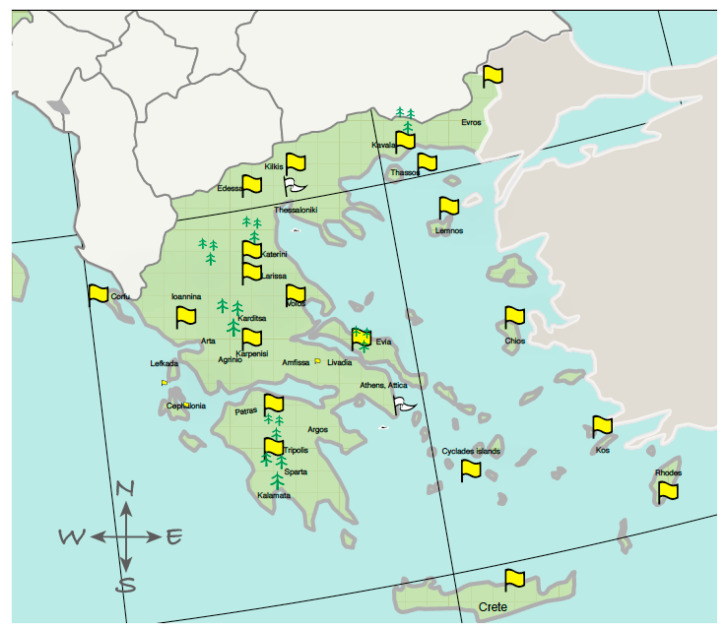
Sample collection points., created by Ortelius software Version 2.0.7.

Honey, pollen, and beebread samples of the present study were collected from different areas in Greece during 2015–2020 (Figure 1). Individual beekeepers and public authorities proceeded to sample in the context of monitoring pesticide residues in these commodities related, in some cases, to bee intoxication incidents. After field sampling, the samples were sent to the lab to investigate the occurrence of pesticide residues and metabolites. In some cases, beebread was carefully isolated from beeswax. Due to the limited number of beebread samples, the latter was considered the same matrix with pollen (in total, 63 honey and 46 pollen and beebread were collected). Honey (multifloral honey) used as a control sample was obtained from beehives of organic apiculture origin. Similarly, for pollen, a sample previously checked and devoid of pesticide residues (of the presented scope) was selected as a blank sample.

### 2.2. Chemicals

Several pesticides and metabolites (more than 130 analytes) were monitored in the current study by applying liquid and gas chromatography–tandem mass spectrometry (LC-MS/MS, GC-MS/MS). Analytical standards and materials used are described in previous works of our group [11,15,16,17]. Carbendazim-d3, imidacloprid-d4, dimethoate-d6, and chlorpyrifos-d10 were used as mass-labeled internal standards for the quantification of the compounds measured by LC-MS/MS, and they were purchased from Sigma-Aldrich (Seelze, Germany). Triphenyl phosphate (TPP), deltamethrin-d6, and dichlorvos-d6 were used in the GC-MS/MS analysis and acquired from Sigma-Aldrich (Seelze, Germany), respectively.

### 2.3. Sample Preparation

The sample preparation for the quantitative method applied for the analysis of all the honey, pollen, and beebread samples of the current study was previously described by our group [11,16,17]. Briefly, 1 g of each matrix was spiked with the six internal standards, carbendazim-d3, imidachloprid-d4, dimethoate-d6, chlorpyriphos-d10, deltamethrin-d6, and triphenyl phosphate (TPP). The extraction step was performed by the application of a modified QuEChERS method using C18, PSA, and Z-Sep. The extract was then centrifuged (4500 rpm, 10 min), and the supernatant was collected, and the final extract was divided into two parts. The two aliquots were evaporated till dryness, and then they were reconstituted with 1 mL of a 75:25 (*v*/*v*) MeOH:H_2_O and pure acetone, respectively. The former aliquot was measured in LC-MS/MS, while the second in GC-MS/MS system.

### 2.4. Instrumental Analysis

All the samples were analyzed both in LC-MS/MS and GC-MS/MS. Regarding LC-MS/MS, an Agilent triple quadrupole (6410 QQQ) system was used, and an injection volume of 20 μL was applied. The GC-MS/MS analysis of the samples was performed on a Chromtech Evolution 3 MS/MS triple quadrupole mass spectrometer built on an Agilent 5975 B inert XL EI/CI MSD system using an injection volume of 2 μL. Chromatographic and mass spectrometric conditions are described in the Supplementary Data and also in previous works of our analytical group [11,16,17].

### 2.5. Quantification and Quality Assurance

The two methods (LC-MS/MS and GC-MS/MS) were validated for specificity, selectivity, reproducibility, repeatability, recovery, and sensitivity according to SANTE guidance documents (applicable at the time of the study) on analytical quality control and method validation procedures for pesticide residues analysis in food and feed [18,19]. For analyte confirmation, retention time (RT) and ion ratio were used. Recoveries were estimated using internal standards and were found to vary between 65 and 120% for all the analytes. The repeatability and reproducibility of both methods were tested by the analysis of multiple spiked with the mixture of analytical standards samples at three different concentrations (at LOQ, 10LOQ, and 100LOQ). The calculated limits of quantification LOQs and other validation metrics for each individual compound are presented in the Supplementary Data (Table S1) and in previous works of our group [11,16,17]. Honey and pollen used as control samples were also analyzed to monitor background contamination. Control samples were analyzed in every sequence of samples, and no pesticide residues and their metabolites were detected in any of them.

### 2.6. Human Health Noncarcinogenic Risk Assessment

The risk to human health posed by the pesticides detected in honey and pollen samples was assessed using the Hazard Quotient and Hazard Index approach [20]. The hazard quotient (*HQ*, unitless) was evaluated for each pesticide in honey and pollen, considering the dietary exposure via honey and pollen consumption. The *HQ*s have been computed following Equations (1) and (2), as depicted below:

(1) $$HQ=\frac{ADD}{ADI}\;$$

(2) $$ADD=C\times \frac{IR}{BW}\;$$

where: *ADD* is the average daily pesticide intake (μg·kg^−1^·d^−1^), *ADI* is the acceptable daily intake (or daily reference dose, μg·kg^−1^·d^−1^) set by EFSA (peer review of pesticides risk assessment), *C* is the mean of pesticide concentration in honey and pollen (μg·kg^−1^), *IR* is the daily honey pollen consumption rate (kg·person^−1^·d^−1^), (honey: 0.005 [21]; pollen: 0.02 for children and 0.04 for adults [22,23]), and *BW* is mean body weight (70 kg for adults, and 15 kg for children).

The *ADI* of an active substance (related to hazard identification and characterization) is based on the assessment of accessible toxicological data and is defined after the establishment of the no-observed-adverse-effect level (NOAEL) and use of the appropriate assessment factor. If the *HQ* is ≤1, it indicates that no adverse effect is likely to occur (health-protective). If *HQ* is >1, then a high level of concern is indicated for chronic effect occurrence. The higher the *HQ*, the higher the concern for chronic toxic effects, highlighting the need for immediate risk management actions.

For the estimation of the total risk from the simultaneous exposure to the mixture of chemicals that might be present in the commodity, the hazard index approach (*HI*, unitless) was applied to approximate the overall risk of multiple pesticides. In the specific approach, the hypothesis of dose additivity was assumed and calculated as the summation of individual *HQ* values (Equation (3)):

(3) HI=ΣHQs=HQ1+HQ2+HQ3+…+HQn 

### 2.7. Bee Health Risk Assessment

The risk assessment for bees was based on EFSA’s bee guidance document [24] and the published risk assessment procedure by Sanchez-Bayo and Goka [25]. LD50 values for pesticides were retrieved from EFSA’s publications on active substances and the Pesticide Properties Database of the University of Hertfordshire [26]. 

To address the subsequent risk to bees due to the consumption of contaminated pollen, the standard risk approach was followed, taking into account EFSA’s bee guidance document [24] and a pertinent published work [25]. To determine the risk, the following equation was followed:

(4) $$Risk=F\;\left(\%\right)\times \frac{dose}{LD50}\;$$

where *F*: the % detection frequency of the active substance in samples; *dose*: is the daily dose in μg per bee, considering pollen’s daily consumption by bees (maximum consumption as a worst-case scenario) and concentration (average and maximum) obtained in this work; *LD*50: median lethal dose per bee (oral and contact, in μg per bee). The frequency of detection is essential since it depicts, as mentioned by previous research group [25], the probability of exposure of bees to the determined pollutants.

## 3. Results

### 3.1. Pesticide and Metabolites Residues

**Figure 2:**
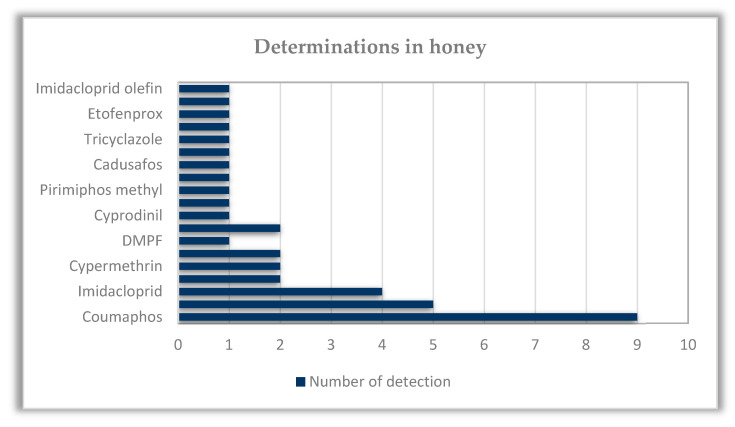
Graph showing determinations of active substances in honey (imidacloprid olefin is a metabolite of imidacloprid).

**Figure 3:**
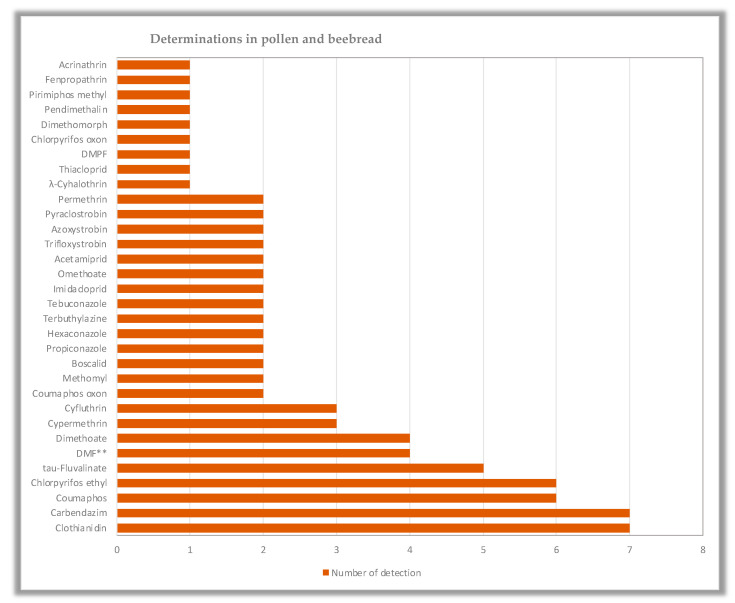
Graph showing determinations of active substances in pollen beebread.

The analytical results showed a 26% positive to at least one active substance honey samples. In these samples, 19 active substances were detected in total, while the most common combination comprised coumaphos (an organophosphorus insecticide and acaricide approved as a veterinary medicinal product [27]), imidacloprid, and DMF (a metabolite of the acaricidal active substance amitraz approved only for veterinary use [27]) (Table 1 and Figure 2). In pollen and beebread, a higher number of active substances were identified (32), accompanied by a superior number of determinations (including a higher number of fungicides detected compared to honey) and an advanced proportion of positive samples (65%) (Table 2 and Figure 3). The latter designates that pollen constitutes a better environmental marker compared to honey, which is reasonable since pollen and nectar are the primary nutrition sources for bees, unsheltered from a plethora of organic and inorganic pollutants [15,16,28].

In honey, MRLs were surpassed for three active substances. More specifically, in one case, coumaphos (as the sum of coumaphos and its metabolite coumaphos oxon) was quantified slightly above its MRL, while the other exceedances were registered for imidacloprid (two cases at 286.8 and 784.7 ng/g) and pirimiphos-methyl (Table 2). In all cases in which exceptionally high concentrations of pesticides encountered were associated with honey samples originating from honeycombs, honeybee death incidents were observed. The latter might postulate misapplications of PPPs, drift phenomena carrying substances away from application fields, applications during bees flying, or even deliberate application of these PPPs to harm the honeybee colonies. It is noteworthy that banned active substances were also detected at quantifiable concentrations. Among them were two organophosphates, cadusafos and ethoprofos, the triazolobenzothiazole active substance tricyclazole, and the pyrethroid cyfluthrin. Nevertheless, for some of the detections in the presented research, we cannot exclude the previously contaminated honeycomb as a potential contributing factor since incoming information from the beekeepers reported long-term use of the same honeycombs. Similarly, MRL exceedances were observed in pollen for clothianidin, coumaphos (the highest concentration observed in one beebread sample), dimethoate, omethoate, tebuconazole, methomyl and pirimiphos-methyl (Table 2).

Βanned pesticides were also detected in pollen and beebread samples. These were fenpropathrin, methomyl, and permethrin. The above results confirm the higher pesticide load of pollen in comparison to the respective levels in honey. Hence, pollen, though far less consumed than honey, deserves noticeable attention, as it is more prone to environmental contaminants. Another valuable conclusion is that pollen is a better marker of environmental contamination of the sampled areas, particularly of pesticides, but also inorganic contaminants, as reported in recent works of our group [15,28].

### 3.2. Human Health Risk Assessment

Prior to commenting on the health risk assessment results, a basic admission on the consumption of pollen was contemplated. Daily consumption of honey is established in the European Union at 5 g per day. On the contrary, due to the rarity of pollen consumption data, published works mostly on therapeutic uses of pollen were considered [22,29] and also embraced in previous work of our group [15]. Yet, an overestimation of the risk due to elevated pollen consumption is expected.

Regarding the health risk assessment, the average daily intake of each pesticide, ADI, and HQ values are presented in Table 3. The only active substance for which the health risk assessment showed (in two samples) alarming levels was coumaphos. More specifically, when mean coumaphos concentration in pollen was incorporated in respective endpoint calculations, it led to an HQ value for children above the threshold value. Similar conclusions were derived when HI values were subtracted.

Considering honey, an exemplary “worst-case” sample contained coumaphos (and coumaphos oxon as the sum, at 101.5 ng/g) accompanied by imidacloprid (286.8 ng/g) and acetamiprid (20.5 ng/g). Nevertheless, after individual calculations of HQs, their summation led to an HI value of 0.04 and 0.13 for adults and children, correspondingly. Consequently, no risk was posed for humans after the potential consumption of this honey.

### 3.3. Bee health Risk Assessment

The risk calculations (Table 4) (see Table S2 for bumblebees and Table S3 for solitary bees) demonstrated negligible risk for bees considering the available consumption data for pollen, including honey bees, bumblebees, and solitary bees [24]. With regard to honeybee consumption, the results refer only to the nurse bee (for foragers, zero consumption is considered).

## 4. Discussion

Multiresidue methods (LC, GC-MS/MS) constitute the mainstay of analytical laboratories involved in pesticide residue analysis in a multitude of matrices. The latter was verified in this work through the detection and quantitation of numerous active substances and metabolites in both pollen beebread and honey. It is important to point out that the presented results are the outcome of the chemical analysis of randomly collected and dispatched honey and pollen samples. Hence, it cannot be viewed under an organized sampling/monitoring scheme in which PPP applications could be straightforwardly connected to specific crops via concomitant monitoring of residues in apicultural commodities, crops, and soil. Nevertheless, based on the incoming information from individual authorities that sent the samples, it was endeavored to proceed to potential associations of active substances detected with the predominant crops in some of the regions and the timing of sampling. In the same context, honey and pollen (and beebread) samples associated with bees’ intoxication incidents (in which deliberate application cannot be excluded) were not included in these interpretations since it would possibly lead to misleading assumptions for PPP applications in the specified areas. A characteristic example stems from one of the few lowland areas of the island region of Chios Island (northern Aegean Sea), in which the cultivation of vegetables and watermelons is documented. From this region, two honey samples were found positive for pesticides. The detection of coumaphos in one sample can be attributed to acaricidal treatments. However, detecting the fungicide active substance penconazole in both honey samples designates the potential uptake of this chemical by bees in the nearby cultivations and subsequent transfer to the beehive, or potential transfer due to pesticides’ drift favored by environmental conditions. Penconazole formulations were approved to control the fungi *Sphaerotheca Fuliginea*, *Erysiphe cichoracearum* in watermelon, and other flowering plant species of the Cucurbitaceae family. Plants of this family, such as watermelon (abundant in the region), are pollinated by bees; therefore, this finding can be a logical hypothesis. In the same samples, the organophosphate fumigant insecticide pirimiphos-methyl was also detected. Its presence seems disconnected from the predominant (active) cultivations of the area, yet, this active substance is usually applied to control pests, such as *Sitophilus granarius* and *Oryzaephilus surinamensis*, of stored seeds. Nevertheless, in the past, the specific region had significant production of wheat, which can interplay in the specific finding through the potential application of pirimiphos-methyl in stored wheat areas. Acetamiprid and etofenprox (both insecticides) detection can be attributed to field applications to combat aphids such as *Macrosiphum euphorbiae* in vegetables, or Lepidoptera such as *Pieris brasiccae* in cabbage and European grapevine moth *Lobesia botrana*. In two additional characteristic samples originating from Northern Greece (Chalkidiki region), 11 active substances and metabolites (chlorpyrifos ethyl, carbendazim, dimethoate, omethoate, tebuconazole, trifloxystrobin, acetamiprid, pyraclostrobin, azoxystrobin, pirimiphos-methyl, and fenpropathrin) in total were identified in two samples of pollen (9 and 10 synchronous detections in the two samples correspondingly). The detection of these compounds can be attributed to PPP applications related to crops of the respective area, such as olives, grapes, and cereals. From the above-mentioned substances, dimethoate (and its metabolite omethoate), along with chlorpyrifos, were authorized at the time of the study and widely used for the control of various pests, such as the olive fruit fly (*Dacus oleae*). The strobilurin fungicides detected are also used in the olive crop and grape for the control of mildew or black rot in grapes. Acetamiprid is a neonicotinoid active substance with a wide range of applications, such as the control of *Lobesia botrana* (major threat to grapes) and *Scaphoideus titanus* in grapes. In addition, the documented substances are applied in crops that bees visit for pollen and nectar, such as stone fruits that are cultivated in the specific region (i.e., peach tree). Consequently, it is possible to rationalize the presented results in the context of land use of the specific regions and PPP applications. For coumaphos and amitraz (via its metabolites detection), their prevalence is expected due to their use as veterinary medicinal products to control the parasitic mite *Varroa destructor*. Last decade’s literature verifies that these compounds are still detected in hive products [30,31,32,33,34], demonstrating in some cases exceedances of the ascribed MRLs (see indicatively [31]). As regards the other detected substances, the findings are largely in line with the recent bibliography. More specifically, in the recent literature concerning honey, pollen, and beebread [35,36,37,38], substances such as boscalid, carbendazim, pyraclostrobin, chlorpyrifos, tau-fluvalinate, cypermethrin, fenpropathrin, and λ-cyhalothrin that were detected in the presented study are also reported. Nevertheless, compounds such as fluopyram and chlorothalonil that are reported in the bibliography were not included in the scope of the presented analytical method.

Regarding the human health risk assessment, the current findings should not be neglected due to the less frequent consumption of pollen/beebread. The last 20-year trend in the consumption of raw, unprocessed food [39], especially of organic origin with proven health benefits, involves apicultural products and is an attitude that steadily increases. The inclination to these products is further strengthened by the scientific reviews on the beneficial effects of the consumption of apicultural commodities on human health [40]. Therefore, it is anticipated that more adults and children are expected to consume such products in all their variations (e.g., pure pollen, beebread, honey, or wax-containing products). Another viewpoint is the consumption of beebread or honeycomb containing it, especially in agricultural communities where the proximity to “raw, unprocessed” food is easier. Honeycombs usually are not devoid of Varroa mites, leading to inevitable applications of coumaphos and other acaricides belonging to the class of organic chemicals. Last but not least, bees’ susceptibility to mixtures of chemicals via pollen consumption is also apparent and can affect their longevity and survival. Consequently, any work or report on residue finding is of utmost importance and should be disseminated to increase awareness for the protection of such a pivotal insect.

## 5. Conclusions

Extensive monitoring of pesticides and their metabolites (due to the broad applications of plant protection products) in apiculture commodities is pivotal due to their consumption by both adults and children and the consumption of pollen by bees. In the same context, monitoring these matrices can unveil, to a certain degree, the contamination that occurs in the agroenvironment. The presented study, after implementing two multiresidue methods, depicted the residual prevalence of pesticides and metabolites in Greek honey and pollen samples between 2015–2020, corroborating the detection of 40 active substances in total (including metabolites) in an overall concentration range of <LOQ to 785 ng/g. Consequently, a risk assessment was conducted to evaluate the potential health effects on humans and bees. The health risk assessment demonstrated, in the majority of cases, negligible risk for bees and humans. Nevertheless, the more than 30 active substances detected in pollen, though anticipated, confirm the occurrence of a plethora of contaminants in an important bee nutrition matrix that deserves attention both from health risk assessment and environmental perspectives.

## Figures and Tables

**Table 1:** Active substances, determinations, and concentration ranges in honey samples.

Active Substance	Determination	Concentration Range (ng/g)	MRL (ng/g)	Authorization at Sampling Period
Coumaphos	9	4.3–**88.7 ^a^**	100	Yes (approved as veterinary medicinal product)
Acetamiprid	5	3.1–20.5	50	Yes
Imidacloprid	4	25.1–**784.7 ^a^**	50	Yes
Coumaphos oxon	2	2.8–**12.8 ^a^**	100	Coumaphos metabolite
Cypermethrin	2	5.1–8.2	50	Yes
DMF *	2	4.9–11.2	200 *	Yes, amitraz metabolite (amitraz approved as veterinary medicinal product)
DMPF *	1	6.9	200 *	Yes, amitraz metabolite
λ-Cyhalothrin	2	4–7.2	50	Yes
Cyprodinil	1	31	50	Yes
Penconazole	1	15.9	50	Yes
Pirimiphos-methyl	1	**53.7 ^a^**	50	Yes
Malathion	1	26.5	50	Yes
Cadusafos	1	1.8	10	No
Ethoprofos	1	1.3	NA	No
Tricyclazole	1	1.4	50	No
Cyfluthrin	1	3.7	50	No **
Etofenprox	1	35	50	Yes
Tau-fluvalinate	1	10.3	50	Yes
Imidacloprid olefin	1	34.5		Imidacloprid metabolite

* Amitraz metabolite, MRL, applies only for its use as veterinary substance; ** β-cyfluthrin was approved, ^a^: in bold, MRL exceedance, for coumaphos it applies after summation with the highest concentration of its metabolite coumaphos oxon.

**Table 2:** Active substances, determinations, and concentration ranges in pollen beebread samples#.

Active Substance	Determination in Pollen Beebread	Concentration Range (ng/g)	MRL (ng/g)	Frequency of Detection (%)
Clothianidin ^a^	7	8.9–136.4 *	50	8.5
Carbendazim ^a^	7	3.4–18	1000	8.5
Coumaphos	6	14.5–511.3 *	100	7.3
Chlorpyrifos ethyl ^a^	6	5.8–35.9	50	7.3
Tau-fluvalinate	5	<LOQ-180	NA	6.1
DMF **	4	4.9–14	200	4.9
Dimethoate ^a^	4	7.9–210 *	10	4.9
Cypermethrin	3	5.9–7.9	50	3.7
Cyfluthrin	3	3.7–11.8	50	3.7
Coumaphos oxon	2	2.2–10.7	100	2.4
Methomyl ^b^	2	85.6–154.6 *	10	2.4
Boscalid ^a^	2	5.3–10.4	50	2.4
Propiconazole	2	31.6–31.9	50	2.4
Hexaconazole ^b^	2	1.3–17.9	NA	2.4
Terbuthylazine ^a^	2	45–53.2	50	2.4
Tebuconazole ^a^	2	7.1–150 *	50	2.4
Imidacloprid ^a^	2	4.5–11.8	50	2.4
Omethoate ^c^	2	13–30 *	10	2.4
Acetamiprid	2	0.9–1.8	50	2.4
Trifloxystrobin ^a^	2	1,6–18	50	2.4
Azoxystrobin ^a^	2	2.1–3.1	50	2.4
Pyraclostrobin ^a^	2	6.6–13	50	2.4
Permethrin ^b^	2	11–30	NA	2.4
λ-Cyhalothrin ^a^	1	7.2	50	1.2
Thiacloprid ^a^	1	172	200	1.2
DMPF **	1	6.9	200	1.2
Chlorpyrifos oxon ^d^	1	9.7	50	1.2
Dimethomorph ^a^	1	15.2	50	1.2
Pendimethalin ^a^	1	10.9	50	1.2
Pirimiphos-methyl	1	170 *	50	1.2
Fenpropathrin ^b^	1	<LOQ	NA	1.2
Acrinathrin ^a^	1	9.9	50	1.2

# For authorization status of common active substances, see Table 1; * in bold, MRL exceedance; ** amitraz metabolite; NA, not available; ^a^ authorized/approved at time of sampling; ^b^ not approved; ^c^ dimethoate metabolite; ^d^ chlorpyrifos ethyl metabolite.

**Table 3:** Human health risk assessment for honey and pollen consumption.

Honey						Pollen					
	Average Daily Intake (μg/kg/day)		ADI (μg/kg/day)	HQ			Average Daily Intake (μg/kg/day)		ADI (μg/kg/day)	HQ	
Active Substance	Adults	Children		Adults	Children	Active Substance	Adults	Children		Adults	Children
Acetamiprid	8.43 × 10^−4^	0.004	25	3.37 × 10^−5^	1.57 × 10^−4^	Boscalid	0.005	0.010	40	1.03 × 10^−4^	5.01 × 10^−4^
Cadusafos	1.29 × 10^−4^	6.00 × 10^−4^	0.4	3.21 × 10^−4^	0.001	Carbendazim	0.006	0.012	20	0.0003	0.0012
Coumaphos *	3.78 × 10^−3^	0.018	0.25	0.015	0.070	Chlorpyrifos *	0.017	0.034	1	0.017	0.068
Cyfluthrin	2.64 × 10^−4^	0.001	10	2.64 × 10^−5^	1.23 × 10^−4^	Clothianidin	0.048	0.097	97	4.28 × 10^−4^	0.001
λ-Cyhalothrin	5.14 × 10^−4^	0.002	2.5	2.06 × 10^−4^	9.60 × 10^−4^	Coumaphos *	0.176	0.352	0.25	0.603	1.408
Cypermethrin	4.93 × 10^−4^	0.002	5	9.86 × 10^−5^	4.60 × 10^−4^	Cyfluthrin	0.005	0.010	10	4.43 × 10^−4^	0.001
Cyprodinil	0.002	0.010	30	7.38 × 10^−5^	3.44 × 10^−4^	λ-Cyhalothrin	0.005	0.010	2.5	0.002	0.004
DMF	5.75 × 10^−4^	0.003	3	1.92 × 10^−4^	8.94 × 10^−4^	Cypermethrin	0.005	0.009	5	7.89 × 10^−4^	0.002
DMPF	4.93 × 10^−4^	0.002	3	1.64 × 10^−4^	7.67 × 10^−4^	Dimethomorph	0.010	0.020	50	1.74 × 10^−5^	4.05 × 10^−4^
Ethoprofos	9.29 × 10^−5^	4.00 × 10^−4^	0.4	2.32 × 10^−4^	0.001	DMF	0.006	0.013	3	0.002	0.004
Tau-fluvalinate	7.36 × 10^−4^	0.003	5	1.47 × 10^−4^	6.87 × 10^−4^	DMPF	0.005	0.009	3	0.001	0.003
Imidacloprid	0.029	0.135	60	4.82 × 10^−4^	2.25 × 10^−3^	Hexaconazole	0.006	0.013	5	0.001	0.003
Malathion	0.002	0.009	30	6.31 × 10^−5^	2.94 × 10^−4^	Methomyl	0.080	0.160	2.5	0.032	0.064
Penconazole	0.001	0.005	30	3.79 × 10^−5^	1.77 × 10^−4^	Propiconazole	0.021	0.042	40	4.54 × 10^−4^	0.001
Pirimiphos-methyl	0.004	0.018	4	9.59 × 10^−4^	0.004	Tebuconazole	0.005	0.009	30	1.35 × 10^−4^	3.16 × 10^−4^
Tricyclazole	1.00 × 10^−4^	4.67 × 10^−4^	30	3.33 × 10^−6^	1.56 × 10^−5^	Terbuthylazine	0.033	0.065	4	0.008	0.016
Etofenprox	5.57 × 10^−4^	0.003	30	1.86 × 10^−5^	8.67 × 10^−5^	Thiacloprid	0.115	0.229	10	0.010	0.023
						Imidacloprid	0.015	0.030	60	2.11 × 10^−4^	4.92 × 10^−4^
						Pendimethalin	0.007	0.015	125	4.98 × 10^−5^	1.16 × 10^−4^
						Dimethoate	0.012	0.024	1	0.012	0.024

* Coumaphos oxon and chlorpyrifos oxon concentrations are added to the parent compounds’ concentration (for concomitant detections with the parent). DMF and DMPF are presented for the cases in which separate detections were observed. Their summation has negligible effect on the HQ (in cases in which both were detected).

**Table 4:** Risk calculations for honeybees based on the active substances (mean and maximum concentrations) quantified in pollen samples#.

			Oral		Contact	
	LD50 (Oral, μg per Bee)	LD50 (Contact, μg per Bee)	RISK (Mean)	RISK (Maximum)	RISK (Mean)	RISK (Maximum)
Clothianidin	0.004	0.044	0.019	0.035	0.002	0.003
Carbendazim	100	50	1.09 × 10^−7^	1.84 × 10^−7^	2.19 × 10^−7^	3.69 × 10^−7^
Coumaphos	na	100	-	-	2.31 × 10^−6^	4.49 × 10^−6^
Chlorpyrifos ethyl	0.15	0.068	1.22 × 10^−4^	2.10 × 10^−4^	2.69 × 10^−4^	4.64 × 10^−4^
Tau-fluvalinate	12.6	12	5.26 × 10^−6^	1.04 × 10^−5^	5.51 × 10^−6^	1.10 × 10^−5^
Amitraz (sum of DMF + DMPF)	na	50	-	-	1.51 × 10^−7^	2.45 × 10^−7^
Dimethoate	0.1	0.1	6.38 × 10^−4^	1.23 × 10^−4^	6.38 × 10^−4^	6.38 × 10^−4^
Cypermethrin	0.172	0.023	1.76 × 10^−5^	2.02 × 10^−5^	1.32 × 10^−4^	1.51 × 10^−4^
Cyfluthrin	0.05	0.001	6.80 × 10^−5^	1.04 × 10^−4^	0.003	0.005
Methomyl	0.28	0.16	1.26 × 10^−4^	1.62 × 10^−4^	2.20 × 10^−4^	2.83 × 10^−4^
Boscalid	160	200	1.44 × 10^−8^	1.90 × 10^−8^	1.15 × 10^−8^	1.52 × 10^−8^
Propiconazole	100	100	9.29 × 10^−8^	9.34 × 10^−8^	9.29 × 10^−8^	9.34 × 10^−8^
Hexaconazole	100	na	2.81 × 10^−8^	5.24 × 10^−8^	-	-
Terbuthylazine	22.6	32	6.36 × 10^−7^	6.89 × 10^−7^	4.49 × 10^−7^	4.87 × 10^−7^
Tebuconazole	83.05	200	2.77 × 10^−7^	5.29 × 10^−7^	1.15 × 10^−7^	2.19 × 10^−7^
Imidacloprid	0.0037	0.081	6.45 × 10^−4^	9.33 × 10^−4^	2.95 × 10^−5^	4.26 × 10^−5^
Acetamiprid	14.53	8.09	2.72 × 10^−8^	3.63 × 10^−8^	4.88 × 10^−8^	6.51 × 10^−8^
Trifloxystrobin	200	200	1.43 × 10^−8^	2.63 × 10^−8^	1.43 × 10^−8^	2.63 × 10^−8^
Azoxystrobin	25	200	3.04 × 10^−8^	3.63 × 10^−8^	3.80 × 10^−9^	4.54 × 10^−9^
Pyraclostrobin	110	100	2.61 × 10^−8^	3.46 × 10^−8^	2.87 × 10^−8^	3.80 × 10^−8^
Permethrin	0.13	0.024	4.61 × 10^−5^	6.75 × 10^−5^	2.50 × 10^−4^	3.66 × 10^−4^
λ-Cyhalothrin	0.91	0.038	1.16 × 10^−6^	1.16 × 10^−6^	2.77 × 10^−5^	2.77 × 10^−5^
Thiacloprid	17.32	38.82	1.45 × 10^−6^	1.45 × 10^−6^	6.48 × 10^−7^	6.48 × 10^−7^
Dimethomorph	32.4	102	6.86 × 10^−8^	6.86 × 10^−8^	2.18 × 10^−8^	2.18 × 10^−8^
Pendimethalin	na	100	-	-	1.95 × 10^−8^	1.59 × 10^−8^
Pirimiphos-methyl	0.22	na	1.13 × 10^−4^	1.13 × 10^−4^	-	-
Fenpropathrin	na	0.05	-	-	2.93 × 10^−6^	2.93 × 10^−6^
Acrinathrin	0.077	0.084	1.88 × 10^−5^	1.88 × 10^−5^	1.72 × 10^−5^	1.72 × 10^−5^

# For the combinations DMF + DMPF (DMA was not detected), chlorpyrifos + chlorpyrifos oxon, and coumaphos + coumaphos oxon, concomitant determinations were considered the sum for risk assessment; na, not available in the open literature.

## Data Availability

All data available are presented in this manuscript.

## Supplementary Materials

The following supporting information can be downloaded at: https://www.mdpi.com/article/10.3390/foods12040706/s1, Text: chromatographic and mass spectrometric conditions, Table S1: active substances and LOQs (pollen and honey), Table S2: risk calculations for bumble bees based on the active substances (mean and maximum concentrations) quantified in pollen samples, Table S3: risk calculations for solitary bees based on the active substances (mean and maximum concentrations).
