# Supplementary material for: Pesticide Residues and Metabolites in Greek Honey and Pollen: Bees and Human Health Risk Assessment
Source: Foods. 2023 Feb 6;12(4):706. doi: 10.3390/foods12040706 (PMC9955768; doi:10.3390/foods12040706)
Supplement: Supplementary file 1 [file foods-12-00706-s001.zip › foods-2161755-supplementary.pdf]

SUPPLEMENTARY DATA to the paper entitled:

Pesticide Residues and Metabolites in Greek Honey and Pollen: Bees and Human Health Risk Assessment

Konstantinos M. Kasiotis,* Effrosyni Zafeiraki, Electra Manea-Karga, Pelagia Anastasiadou and Kyriaki Machera

Benaki Phytopathological Institute, Laboratory of Pesticides' Toxicology, 8 St. Delta Street, Athens, Kifissia 14561, Greece

To whom correspondence should be addressed. E-mail: k.kasiotis@bpi.gr

This document provides more detailed information to the main paper mentioned above. The following information includes:

**Content**		**Page**
Text	Chromatographic and mass spectrometric conditions	S1
Table S1	Active substances and LOQs (pollen and honey)	S2
Table S2	Risk calculations for bumble bees based on the active substances (mean and maximum concentrations) quantified in pollen samples	S3
Table S3	Risk calculations for solitary bees based on the active substances (mean and maximum concentrations) quantified in pollen samples.	S4

Text

Chromatographic and mass spectrometric conditions

LC-ESI-MS/MS

For the determination of pesticide residues in honey and pollen samples an LC-ESI-MS/MS was used, as described in detail in preceding publications of our group (Kasiotis et al., 2018, 2021; Zafeiraki et al., 2022). Briefly, using a typical C18 column, the chromatographic gradient started from 80% 5 mM ammonium formate in H_2O, 0.1% formic acid (mobile phase channel A) to 100% 5 mM ammonium formate in MeOH, 0.1% formic acid (mobile phase channel B) in 30 min. Each chromatographic separation lasted 35 min and the injection volume was 20 μL. Furthermore, the flow rate was set at 0.3 mL/min, while the analytical column was thermostated at 30 °C. The analyses were performed with a multiple reaction monitoring (MRM) method monitoring two mass transitions for every analyte.

GC-MS/MS

The GC-MS/MS analysis of the samples was performed on a Chromtech Evolution 3 MS/MS triple quadrupole mass spectrometer built on an Agilent 5975 B inert XL EI/CI MSD system. The chromatography system was equipped with a Gerstel MPS-2 autosampler and a HP-5ms UI column (length 30m, ID 0.25mm, film thickness 0.25 μm, J&W Folsom, USA). For the separation of the analytes, the followed chromatographic gradient was applied: initial oven temperature of 50 °C for 1min, increase of temperature till 125 °C at a rate of 25 °C/min and finally reaching 300 °C at a rate of 10 °C/min. The analyses in MS/MS were performed with a multiple reaction monitoring (MRM) method that monitored two mass transitions (parent ion/product ion) for every analyte, while Argon 99.9999% was used as a collision gas. The electron multiplier voltage was set at 2000 V and the duration of MS/MS measurement was 36.5 min for each sample.

Table S1: Active substances and LOQs (pollen and honey)

Analyte	LOQ ng/g	Analyte	LOQ ng/g	Analyte	LOQ ng/g
Acetamiprid	1	EPN	10	Penconazole	5
Aclonifen	10	Ethion	10	Pencycuron	10
Alachlor	0.5	Ethoprophos	0.5	Pendimethalin	10
Aldicarb sulfone	5	Famoxadone	0.5	Phosalone	5
Ametryn	0.5	Fenamidone	5	Phosmet	1
Atrazine	10	Fenamiphos	5	Phosphamidine	5
Azimsulfuron	1	Fenamiphos sulfone	1	Phoxim	5
Azoxystrobin	0.5	Fenamiphos sulfoxide	1	Piperonyl butoxide	5
Benalaxyl	5	Fenbuconazole	1	Pirimicarb	5
Benfuracarb	1	Fenpropathrin	10	Pirimiphos-methyl	1
Bensulfuron-methyl	1	Fenpropimorph	10	Prometryn	0.5
Benfluralin	10	Fenthion	10	Propiconazole	5
Boscalid	5	Fenthion-sulfone	10	Prothioconazole desthio	5
Bupirimate	5	Fenthion-sulfoxide	1	Pyraclostrobin	5
Buprofezin	5	Flusilazole	1	Pyrazophos	1
Cadusafos	1	Fosthiazate	1	Pyrimethanil	5
Carbendazim	1	Haloxyfopethyl ester	5	Quinoxyfen	5
Carbofuran	1	Imazalil	5	Spinosad A	0.5
Carboxin	1	Imidacloprid	1	Spiroxamine	0.5
Chlorpyrifos ethyl	5	Indoxacarb	1	Tebuconazole	5
Chlorsulfuron	5	Iprodione	5	Tebufenozide	5
Clethodim	5	Iprovalicarb	1	Terbufos	5
Clofentezine	5	Isoproturon	1	Terbuthylazine	5
Clothianidin	1	Linuron	0.5	Tetraconazole	10
Chloridazon	1	Malaoxon	5	Thiabendazole	1
Cyproconazole 1	5	Malathion	5	Thiacloprid	0.5
Cyproconazole 2	5	Mepanipyrim	5	Thiamethoxam	0.5
Cyprodinil	5	Metalaxyl	0.5	Thifensulfuron-methyl	5
Demeton-S-methyl	0.5	Metconazole	0.5	Thiodicarb	1
Demeton-S-methyl sulfoxide	5	Methidathion	1	Triazophos	1
Desmetryn	1	Methoxyfenozide	0.5	Tricyclazole	1
Diazinon	10	Metoxuron	5	Trifloxystrobin	1
Diethofencarb	5	Metribuzin	1	Triticonazole	10
Difenoconazole	1	Monocrotophos	0,5	Triadimefon	10
Dimethoate	1	Monolinuron	1	Zoxamide	5
Dimethomorph	5	Myclobutanil	5	Coumaphos	1
Disulfoton	5	Nicosulfuron	1	Cexaconazole	10
Disulfoton sulfoxide	10	Omethoate	1	Cenazaquin	10
Dodemorph	1	Oxadiazon	1	Propargite	5
Etoxazole	1	Paraoxon-methyl	10	Methomyl	10
Propamocarb	5	Fludioxonil	5	Amitraz	5
Napropamide	1	DMF	5	DMPF	5
DMA	10	Etofenprox	1	Cypermethrin	5
Deltamethrin	5	Coumaphos oxon	5	Chlorpyrifos oxon	5

Hexaconazole	1	Acrinathrin	5		

Table S2. Risk calculations for bumble bees based on the active substances (mean and maximum concentrations) quantified in pollen samples[a].

			Oral		Contact	
**Active substance**	**LD50 (oral, μg per bee)**	**LD50 (contact, μg per bee)**	**RISK (mean)**	**RISK (maximum)**	**RISK (mean)**	**RISK (maximum)**
Clothianidin	na	0.02	-	-	0.0094	4.52E-06
Chlorpyrifos ethyl	0.23	1.58	2.01E-04	3.46E-04	2.93E-05	2.74E-12
tau-Fluvalinate	31.3	na	5.34E-06	1.06E-05	-	-
Dimethoate	0.33	0.94	4.88E-04	9.41E-04	1.71E-04	3.96E-11
Cypermethrin	0.263	0.119	2.91E-05	3.33E-05	6.43E-05	1.18E-12
Cyfluthrin	0.11	0.36	7.81E-05	1.19E-04	2.39E-05	8.72E-14
Imidacloprid	0.038	0.218	1.59E-04	1.34E-04	2.76E-05	1.34E-04
Acetamiprid	22.2	100	4.49E-08	7.36E-09	9.98E-09	7.36E-09
Pyraclostrobin	97.2	100	7.45E-08	3.86E-07	7.24E-08	3.86E-07
Permethrin	na	0.22	-	-	6.89E-05	8.47E-04
λ-Cyhalothrin	0.16	0.11	1.66E-05	1.43E-04	2.42E-05	1.43E-04
Thiacloprid	na	100	-	-	6.35E-07	8.96E-05
Dimethomorph	283.2	300	1.98E-08	2.33E-07	1.87E-08	2.33E-07
Methomyl	>3.3	na	2.69E-05	3.46E-05	-	-

[a]pollen daily consumption 30.3 mg

Table S3. Risk calculations for solitary bees based on the active substances (mean and maximum concentrations) quantified in pollen samples[a].

			Oral		Contact	
	LD50 (oral, µg per bee)	LD50 (contact, µg per bee)	RISK (median)	RISK (maximum)	RISK (median)	RISK (maximum)
Clothianidin	8.4[1]	0.1[1]	7.53E-06	1.41E-05	6.33E-04	0.0012
Chlorpyrifos ethyl	na	4.19[1]	-	-	3.71E-06	6.39E-06
Dimethoate	0.25[2]	1.02[2]	2.17E-04	4.18E-04	5.31E-05	1.02E-04
Propiconazole	>33.3[2]	na	2.37E-07	2.38E-07	-	-
Imidacloprid	na	0.031[1]	-	-	6.54E-05	9.47E-05
Acetamiprid	na	1.72[1]	-	-	1.95E-07	2.60E-07
λ-Cyhalothrin	na	0.136[1]	-	-	6.58E-06	6.58E-06
Thiacloprid	na	1.16[1]	-	-	1.84E-05	1.84E-05

[a]pollen daily consumption of 10.2 mg, [1]: Genus: Osmia bicornis, [2]: Genus: Osmia lignaria
